# Laser Deflection Acoustic Field Quantification: A Non-Invasive Measurement Technique for Focused Ultrasound Field Characterization

**DOI:** 10.3390/bioengineering13010022

**Published:** 2025-12-26

**Authors:** Yang Xu, Hongde Liu, Yaoan Ma, Xiaoxue Bai, Qiangwei Hu, Yunpiao Cai, Hui Zhang, Tao Huang, Mengmeng Liu, Jing Li, Mingyue Ding, Ming Yuchi

**Affiliations:** 1 Department of Biomedical Engineering, School of Life Science and Technology, and Advanced Biomedical Imaging Facility, Huazhong University of Science and Technology, Wuhan 430074, China; d202380996@hust.edu.cn (Y.X.); d202380973@hust.edu.cn (H.Z.); 2 Hubei Medical Devices Quality Supervision and Test Institute, Wuhan 430075, China; baixiaoxue@whmit.cn (X.B.); htao2010@126.com (T.H.); liumengmeng@whmit.cn (M.L.); lijing@whmit.cn (J.L.); 3 School of Optical and Electronic Information, Huazhong University of Science and Technology, Wuhan 430074, China; liuhd@hust.edu.cn (H.L.); d202581662@hust.edu.cn (Y.M.); estherhh@hust.edu.cn (Q.H.); m202473604@hust.edu.cn (Y.C.)

**Keywords:** focused ultrasound, acoustic field reconstruction, laser deflection, non-invasive measurement

## Abstract

Focused ultrasound (FU) technology is extensively employed in clinical applications such as tumor ablation, Parkinson’s disease treatment, and neuropathic pain management. The safety and efficacy of FU therapy critically depend on the accurate quantification of the acoustic field, particularly the high-pressure distribution in focal region. To address the limitations of existing acoustic measurement techniques—including invasiveness, inability to measure high sound pressure, and system complexity—this study proposes a non-invasive method termed Laser Deflection Acoustic Field Quantification (LDAQ), based on the laser deflection principle. An experimental system was constructed utilizing the acousto-optic deflection effect, which incorporates precision displacement control, rotational scanning, and synchronized triggering. Through tomographic scanning, laser deflection images of the acoustic field were acquired at multiple orientations. An inversion algorithm using Radon transforms was proposed to reconstruct the refractive index gradient distributions from the variations of light intensity and spot displacement. An adaptive weighted fusion strategy was then employed to map these optical signals to the sound pressure field. To validate the LDAQ technique, an acoustic field generated by an FU transducer operating at 0.84 MHz was measured. The reconstructed results were compared with both hydrophone measurements and numerical simulations. The findings demonstrated high consistency among all three results within the focal zone. Full-field analysis yielded a root mean square error (RMSE) of 0.1102 between LDAQ and simulation, and an RMSE of 0.1422 between LDAQ and hydrophone measurements. These results confirm that LDAQ enables non-invasive and high-precision quantification of megapascal-level focused acoustic fields, offering a reliable methodology for acoustic field characterization to support FU treatment optimization and device standardization.

## 1. Introduction

Focused Ultrasound (FU) technology, as a non-invasive therapeutic modality, demonstrates significant application potential in biomedical fields such as tumor ablation [1,2,3], neuromodulation [4,5,6], and targeted drug delivery [7,8,9]. Tang et al. [10] utilized FU to induce immunogenic cell death in tumor cells while simultaneously suppressing the immunosuppressive tumor microenvironment. Mekers et al. [11] reported the effects of FU on soluble immunoregulatory factors and immune cells across various preclinical models. Wang et al. [12] evaluated the clinical and immunological effects of FU on colorectal cancer (CRC) liver metastases, demonstrating that FU significantly improved overall survival in patients with CRC liver metastases and reduced serum IL-6 levels. Mast et al. [13] proposed a method for bulk ablation of soft tissue using high-intensity ultrasound, applying FU to soft tissue repair. Han et al. [14] summarized the development of ultrasound-responsive smart composite biomaterials in tissue repair, highlighting their broad applications in anti-infection, skin repair, osteogenesis, gene transfection, thrombolysis, neuromuscular function, and imaging.

The extensive clinical application of focused ultrasound (FU) makes treatment safety a critical prerequisite and core challenge. Existing studies have focused intensively on this area: Fan et al. [15] evaluated the efficacy and safety of FU for treating benign thyroid nodules; Wu et al. [16] systematically analyzed the clinical safety and efficacy of ultrasound-guided HIFU for treating breast fibroadenomas; Yang et al. [17] explored the therapeutic efficacy and safety of HIFU ablation for patients with colorectal liver metastases (CRLM) unsuitable for liver resection.

However, the safety and efficacy of FU therapy fundamentally depend on the precision and reliability of acoustic field parameter measurements [18]. Therefore, precise, non-invasive quantitative characterization of key parameters in the FU acoustic field—particularly sound pressure and intensity at the focal region—is crucial for optimizing clinical treatment protocols, ensuring patient safety, supporting scientific regulation of medical devices, and advancing standardized development of this technology.

Numerous scholars have conducted extensive research in acoustic field quantification. For instance, Janget al. [19] employed hydrophones to measure the radial velocity and distance of a moving sound source, and cross-validated their findings with SWellEx-96 experimental data to enable the estimation of sound source trajectory parameters at extended ranges. Colom, M. et al. [20] achieved rapid quantification of low-pressure ultrasound fields using interferometric wavefront sensing, yielding results highly consistent with hydrophone measurements. The aforementioned studies have provided relatively comprehensive characterization of low-pressure, divergent acoustic fields.

However, existing acoustic field quantification techniques still exhibit significant limitations: hydrophones [21] represent an invasive measurement method and are prone to damage in high-sound-pressure environments (particularly at the focal point of focused ultrasound); interferometry [22] offers high precision but involves complex systems that are difficult to deploy, hindering the practical application; and schlieren-wavefront sensing requires a complex system with optical components of extremely high machining precision, resulting in high cost.

In contrast, laser deflection [23] offers a non-invasive, structurally simple, and cost-effective sound field quantification technique. Previous work by Xue Bin’s team at Tongji University [24] successfully applied this method to quantify sound pressure fields in the kilopascal range, yielding the results highly consistent with simulation data and providing experimental support for Kirchhoff’s integral theorem. Based on this foundation, this study proposes a new non-invasive quantification technique for focused acoustic fields based on laser deflection, aiming to achieve precise measurements of sound pressure fields in megapascal range.

In this study, we introduce and validate the Laser Deflection Acoustic Field Quantification (LDAQ) method, which advances the state of the art in several key aspects: (1) It provides a fully non-invasive measurement solution; (2) By leveraging a tomographic scanning approach combined with a Radon-transform-based inversion algorithm, it enables the reconstruction of refractive index gradient distributions from coupled optical signals (intensity change and spot displacement); (3) The system design emphasizes practicality, employing relatively simple optics and precision motion control to achieve robust operation; (4) the method is experimentally demonstrated to accurately quantify megapascal-level sound pressure fields in the focal zone of a focused ultrasound transducer thereby providing a viable optical-based alternative for the non-invasive characterization of therapeutic ultrasound fields. This work thus establishes LDAQ as a promising tool for the precise, non-invasive characterization of FU fields, with direct relevance to treatment optimization and device standardization.

## 2. Materials and Methods

### 2.1. Theoretical Foundation of Laser Deflection Method for Acoustic Field Inversion

#### 2.1.1. Acousto-Optic Deflection Phenomenon

When sound waves propagate through a medium, they induce periodic perturbations in the medium’s density, leading to the corresponding changes in its refractive index distribution. As a laser beam traverses this medium field with varying refractive index, its wavefront experiences differing degrees of phase delay in regions of varying density, causing the propagation path to deflect. This macroscopic beam deflection phenomenon is known as the acousto-optic deflection effect. Theoretical studies further confirm a direct quantitative relationship between the beam deflection angle and the local sound pressure and sound pressure gradient within the acoustic field. This relationship establishes the theoretical basis for optically inverting the acoustic field distribution.

#### 2.1.2. Derivation of Formulas

In the acousto-optic deflection effect, the change in medium refractive index (Δ*n*) exhibits a linear relationship with local sound pressure (*p*). This relationship can be quantitatively described by the following formula [24]:

(1) $$n\left(r,t\right)=n_{0}+\left(\frac{\delta n}{\delta p}\right)p\left(x,y,z,t\right),$$

where n(r,t) represents the refractive index of the medium; r denotes the spatial coordinate vector; $n_{0}$ is the initial refractive index of the medium; $p\left(x,y,z,t\right)$ characterizes the sound pressure at spatial coordinates at the time t. When a laser beam traverses this medium with varying refractive index, its propagation path undergoes deflection. The relationship between the total deflection angle of the beam and the refractive index gradient can be determined by the following path integral:

(2) $$\alpha =\frac{1}{2n_{0}}\left(\frac{\delta n}{\delta p}\right)\int \frac{\delta p(x,y,z,t)}{\delta z}dx,$$

Theoretical analysis indicates that the relative rate of change in the central intensity of the detection beam is proportional to the cumulative effect of the refractive index gradient along the beam path. Simultaneously, the lateral displacement of the spot position is also proportional to the integral of the refractive index gradient in the propagation direction. Therefore, the relative intensity change ($\Delta I/I_{0}$) and spot displacement (δ) are coupled with the refractive index gradient (∇n) through the following relationship. Among these, (L) represents the path of light propagation.

(3) $$\frac{\Delta I}{I_{0}}=\frac{I_{0}-I}{I_{0}}≅\left|\int_{0}^{L}\nabla ndz\right|,$$

(4) $$R=n(\vec{V}(x,y)+\vec{v}(z))=n\frac{d\vec{r}}{dz}≅\int_{0}^{L}\nabla ndz,$$

Based on the Radon transform, we performed backprojection reconstruction using two sets of projection data: the intensity change rate and the spot displacement. This yielded preliminary refractive index gradient distributions, denoted as (derived from intensity changes) and (derived from displacements). To achieve optimal reconstruction, and were ultimately weighted and fused to obtain high-precision acoustic field reconstruction results.

(5) $$s_{1}(x,y)=\frac{1}{4\pi^{2}}\int_{0}^{2\pi }\int_{0}^{+\infty }\hat{\Delta I}(\omega ,\alpha )e^{i\omega (zcos\alpha +xsin\alpha )}\omega d\omega d\alpha ,$$

(6) $$s_{2}(x,y)=\frac{1}{4\pi^{2}}\int_{0}^{2\pi }\int_{0}^{+\infty }\hat{R}(\omega ,\alpha )e^{i\omega (zcos\alpha +xsin\alpha )}\omega d\omega d\alpha ,$$

The final sound field reconstruction result (s) is obtained by performing a weighted sum of $s_{1}$ and $s_{2}$.

(7) $$s=w_{1}s_{1}+w_{2}s_{2}$$

where $w_{1}$ and $w_{2}$ are weighting parameters that balance the contributions from the two complementary physical effects. These parameters were determined via an iterative optimization process to achieve the best agreement with a reference acoustic field. Specifically, a reference field was generated using the numerical simulation model described in Section 2.2.3. A grid search was then performed over the feasible weight space ($w_{1}+w_{2}=1$, $w_{1},w_{2}\geq 0$) with a step size of 0.01 For each candidate pair, the fused field s was calculated, and its Root Mean Square Error (RMSE) relative to the reference field within the focal region was computed. The weight pair that minimized this RMSE was selected for all subsequent reconstructions presented in this work.

### 2.2. Experimental Setup

#### 2.2.1. Description of the LDAQ System

**Figure 1:**
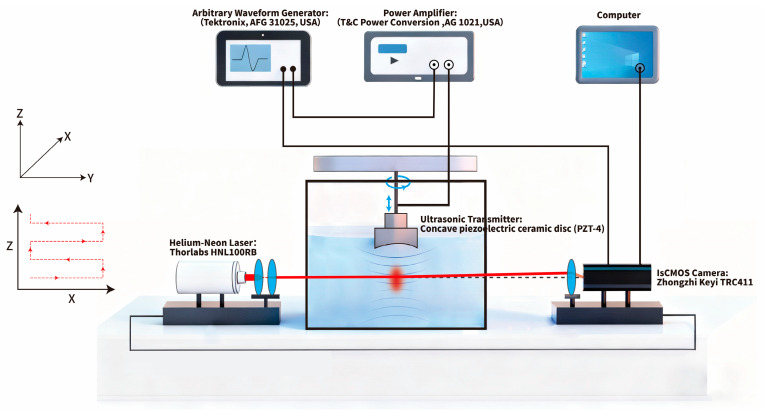
Schematic Diagram of LDAQ Device and Principle.

The schematic diagram of the experimental setup used in this study is shown in Figure 1. A continuous laser beam emitted by a helium-neon laser (Thorlabs HNL100RB, Thorlabs Inc., Newton NJ, USA, output power 10 mW, wavelength λ = 632.8 nm) first passes through a lens assembly for beam expansion and collimation. This lens assembly consists of a plano-convex lens with a radius of curvature of 19.69 mm and a collimating lens with a radius of curvature of 26.25 mm, equipped with a Daheng Optoelectronics GCC-3011A filter(Daheng Optoelectronics, Guangdong, China). The entire lens assembly is precisely integrated into a high-precision three-axis (XYZ) displacement stage to enable precise adjustment of the beam’s collimation state and size.

To enhance system flexibility, we designed a turret-style lens-switching assembly integrating multiple collimating lenses with varying focal lengths. Rotating the turret enables rapid lens switching, facilitating adaptation to different spatial resolution and measurement range requirements.

The collimated beam passes through a custom water bath filled with deionized and deaerated water, interacting with the ultrasonic field within. During the experiments, the water temperature was consistently maintained at 25.0 ± 0.5 °C, and the dissolved oxygen content in the water was kept at 2.4 ± 1 mg/L throughout. The acoustic field induces refractive index variations in the water medium, causing beam deflection. Finally, the modulated output beam is captured and recorded by a high-resolution IsCMOS camera (TRC411, Zhongzhi KeYi, Beijing, China). The laser, water tank, and IsCMOS camera are all mounted on a precision optical vibration isolation platform to ensure mechanical stability and optical path stability of the system.

The ultrasonic emitter employs a concave piezoelectric ceramic transducer (PZT-4, Chongqing Haifu Medical Technology Co., Ltd., Chongqing, China) with a diameter of 50 mm, a curvature radius of 50 mm, and a resonance frequency of 0.84 MHz. Acoustic coupling is achieved by filling the front end of the transducer with a convex polyacrylamide hydrogel (CKC 100, Chongqing Haifu Medical Technology Co., Ltd., Chongqing, China) having a curvature radius of 40 mm. The focal domain dimensions of this transducer are approximately 3.0 mm × 3.0 mm × 35 mm, with a focal plane distance of 46 mm. In the experiment, the drive signal was generated by an arbitrary waveform generator (Tektronix AFG 31025, Tektronix, Inc., Beaverton OR, USA) with the following parameters: 500 mVpp voltage, 30 cycles, and a 10 ms pulse repetition interval. This signal was amplified by a power amplifier (BSA 0125-75, BONN Elektronik, Bonn, Germany, 9 kHz–250 MHz) at a fixed gain of 85% to drive the ultrasonic transducer and generate a focused acoustic field.

The transducer is mounted on a multi-degree-of-freedom adjustment mechanism. This mechanism incorporates a lifting unit for precise control of the transducer’s immersion depth in the water tank, along with a high-precision rotary platform (ZNC05-130-SV-70, DongGuan YiHeDa Automation Co., Ltd., Dongguan, China, positioning accuracy ±30 arcseconds) to drive the transducer through 360° rotation around its axis. Beneath the rotation platform is a dedicated centering unit equipped with a scale and fine-adjustment bolts. This ensures rapid and precise positioning of the transducer at the rotation center, guaranteeing stable rotation of the acoustic field around its center during tomographic data acquisition.

To achieve “instantaneous” ultrasound measurement and precisely locate the acoustic field center, the system employs a sophisticated synchronous triggering mechanism. An arbitrary waveform generator simultaneously sends synchronous trigger signals to both the ultrasonic transducer and the IsCMOS camera. By precisely adjusting the delay between ultrasonic triggering and camera acquisition, the system ensures accurate capture of the peak pressure moment of the ultrasonic pulse within the camera’s extremely short 100 ns exposure time. Since this exposure time is significantly shorter than the ultrasonic cycle, the system effectively “freezes” the acoustic field, yielding an instantaneous beam deflection image corresponding to the peak pressure moment.

#### 2.2.2. Acoustic Field Scanning Process

To achieve acoustic field reconstruction, this study employs a laser-deflection-based tomographic acquisition method. This process enables high-throughput projection data collection through high-precision electromechanical control and synchronized timing triggers. Specifically, a program-controlled rotating platform performs a scanning rotation within a 180° range at fixed 1° increments. At each rotational angle, the displacement stage drives the beam to perform a 30 × 30-point two-dimensional planar scan (with 0.1 mm increments along both X and Z axes) across the entire theoretical focal region. Ultimately, the IsCMOS camera captures 180 × 30 × 30 images throughout the scanning process, forming the complete raw dataset for subsequent tomographic reconstruction.

#### 2.2.3. Simulation

To validate the effectiveness and accuracy of the LDAQ method, this study developed an acoustic-optical coupling simulation model based on the COMSOL (COMSOL Multiphysics 6.3) platform to perform numerical simulations of the FU acoustic field. The acoustic field modeling employed the angular spectrum method, with the simulation domain set as a cubic water volume of 3.0 × 3.0 × 35 mm^3^ and a spatial discretization interval of 0.1 mm. The focusing transducer was simplified as a planar piston sound source with a diameter of 20 mm, a center frequency of 0.84 MHz, and a geometric focal length of 60 mm. The acoustic parameters of the water domain were set as follows: sound velocity 1480 m/s, density 1000 kg/m^3^, and sound attenuation coefficient 0.3 dB/(MHz·cm). A sinusoidal excitation with a peak sound pressure of 2 MPa was applied to the source surface. The steady-state sound pressure distribution in three-dimensional space was calculated using angular spectrum propagation. The simulation employed parameters identical to those of the experimental setup, and the resulting sound pressure distribution was normalized for quantitative comparison with the experimental results.

This simplified model, employing a planar piston source with CW excitation, was designed to provide a computationally efficient yet physically sound theoretical benchmark of the focal pressure field. The chosen parameters (piston diameter and focal length) approximate the effective focusing gain and focal spot size of the experimental concave transducer. The use of CW excitation approximates the quasi-steady-state peak pressure field captured by the LDAQ system at the trigger moment during the long pulse.

#### 2.2.4. Hydrophone Measurements

To provide a reference benchmark, this study characterized the experimental ultrasonic field using a needle hydrophone (NHA0200, Precision Acoustics, Dorchester, UK). The core measurement system comprised this hydrophone, a custom-built precision 3D translation stage, and a high-speed data acquisition card (LDI420VSE, Chengdu Jiayi Technology Development Co., Ltd., Chendu, China). Measurements employed a point-by-point scanning strategy: the hydrophone was fixed at the theoretical focal point of the acoustic field, while the ultrasonic transmitter mounted on the three-dimensional translation stage performed the motion. Programmed control of the translation stage enabled three-dimensional movement of the transmitter relative to the fixed hydrophone, thereby scanning the entire target acoustic field. Data acquisition was precisely synchronized with both mechanical motion and ultrasonic excitation. For each displacement step of the translation stage, the acquisition card recorded the instantaneous sound pressure signal from the hydrophone after a preset time delay. This delay ensured temporal alignment between the acquired sound pressure data and the LDAQ measurements, guaranteeing both systems observed the same spatiotemporal state during the acoustic field evolution. The scanning step size is set to 0.1 mm to balance spatial resolution and scanning efficiency. Ultimately, this method yields a series of acoustic pressure time histories at spatial points, enabling the reconstruction of the three-dimensional spatial acoustic pressure distribution of the ultrasonic field.

### 2.3. Sound Field Inversion Algorithm and Data Processing

**Figure 2:**
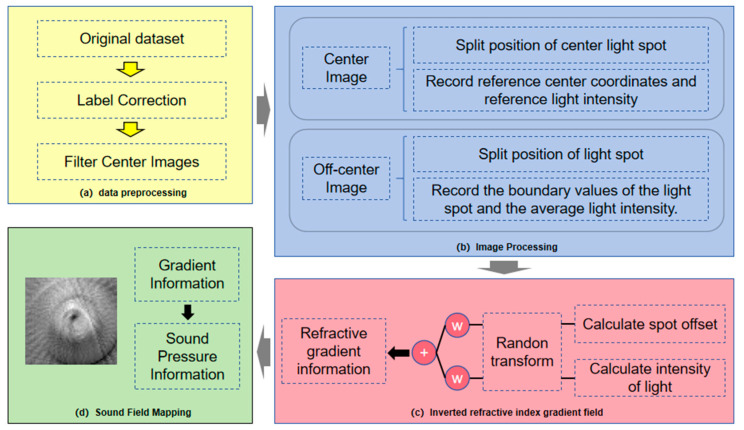
Schematic Diagram of the Acoustic Field Reconstruction Algorithm.

This study developed a refractive index gradient reconstruction method based on optical measurement and computed tomography (CT) technology. By collecting projection data at multiple angles and applying image reconstruction algorithms, this method enables the visualization of refractive index gradient distributions within ultrasonic fields. The algorithm’s specific workflow, illustrated in Figure 2, comprises four main steps: data preprocessing, image processing, refractive index gradient inversion, and acoustic field mapping.

**Figure 3:**
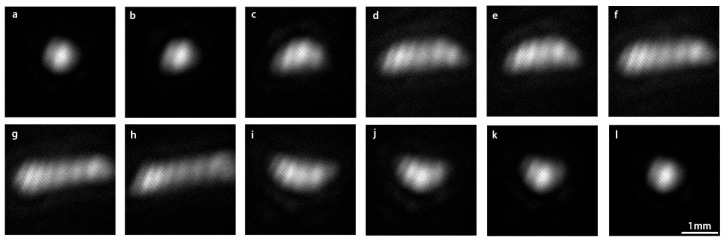
Original experimental images of laser spot displacement captured by the IsCMOS camera within the same period. (**a**–**l**) Images sequentially captured from the edge of the acoustic focal domain with a step interval of 0.5 mm.

During experimental data acquisition, the system precisely controlled the laser incidence angle (range 0° to 180°, 1° increments) and detection position (X-axis: 99.0–105.0 mm; Y-axis: 86.0–104.0 mm) to obtain speckle image datasets under various conditions. A typical speckle displacement image captured by the IsCMOS camera is shown in Figure 3.

During the data preprocessing stage, standardized corrections were applied to the data labels, and reference images centered on the spot positions were extracted. This step aimed to determine the precise coordinate regions of the spots in their undisturbed state, establishing a baseline for subsequent calculations.

During the image processing stage, the Otsu adaptive threshold segmentation algorithm was employed to automatically identify the spot region, precisely extracting the spot’s center coordinates and the average light intensity within that area. For the defined center reference position (x = 99.0 mm, y = 86.0 mm), the reference light intensity and reference center coordinates were calculated for each scanning angle.

The inversion of the refractive index gradient is based on the physical mechanism of acousto-optic interaction, processing two distinct effects represented by the intensity change and the spot displacement. The relationship between the intensity change rate and the refractive index gradient is shown in Equation (3), reflecting the line integral effect of the refractive index gradient along the beam propagation path. The relationship between spot displacement and refractive index gradient is expressed by Equation (4), characterizing the beam deflection caused by the gradient. The specific inversion process is as follows: For each dataset based on intensity and displacement, respectively, extract all X-axis projection data corresponding to the midpoint in the Y-direction across the entire scan plane at each scanning angle. Subsequently, the Radon transform is applied to map these polar coordinate projections onto the Euclidean coordinate plane. Weighted summation is then performed to complete the inversion of the refractive index gradient. This inverted gradient is mapped onto the actual sound pressure field, achieving sound field quantification.

The reconstruction algorithm employs a filter-inverse projection framework. A ramp filter is designed to perform frequency-domain filtering on the projection data, eliminating star artifacts. The two-dimensional distribution of the refractive index gradient is then reconstructed through inverse projection operations. To further enhance reconstruction quality, this study proposes an adaptive weighting fusion strategy. This multimodal fusion method fully leverages the complementary information provided by the two physical effects: light intensity and displacement. The weights for this fusion were determined through a systematic optimization against a simulated reference field, a practical approach to account for the potentially setup-dependent noise characteristics and sensitivity of the two optical signals.

## 3. Results

To systematically validate the reconstruction accuracy of the Laser Deflection Acoustic Field Quantification (LDAQ) technique, this study employed an integrated comparative analysis combining numerical simulations with hydrophone scanning measurements. A numerical model developed using COMSOL Multiphysics software established the acoustic field benchmark based on actual transducer parameters, while a hydrophone controlled by a precision three-dimensional positioning system performed acoustic field scanning measurements in a deionized water environment. These two independent methods provided theoretical reference and experimental verification for acoustic field reconstruction, respectively.

**Figure 4:**
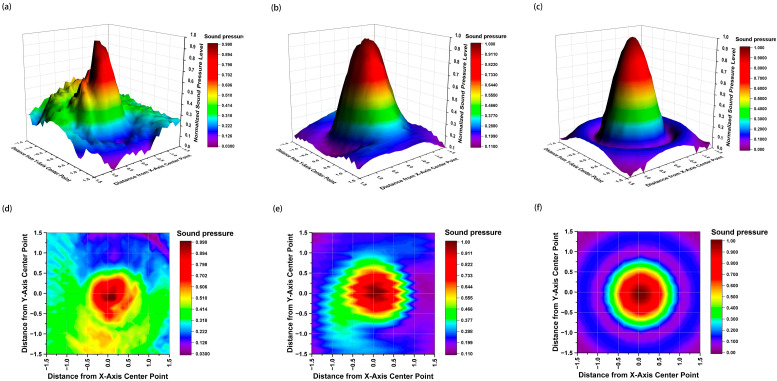
Comparison of Acoustic Field Reconstruction Results with Simulation and Hydrophone Measurements. (**a**) 3D Reconstruction Results of Acoustic Field by LDAQ. (**b**) 3D Measurement Results of Acoustic Field by Hydrophone. (**c**) 3D Simulation Results of Acoustic Field by Numerical Modeling. (**d**) 2D Cross-sectional Distribution of Acoustic Field Reconstructed by LDAQ. (**e**) 2D Cross-sectional Distribution of Acoustic Field Measured by Hydrophone. (**f**) 2D Cross-sectional Distribution of Acoustic Field Simulated by Numerical Modeling.

Figure 4 provides a visual comparison of the spatial distribution of the acoustic field, which demonstrates strong agreement among the three methods. LDAQ successfully reconstructed the three-dimensional structure of the focused acoustic focal zone (Figure 4a) and its corresponding two-dimensional cross-sectional distribution (Figure 4d). The core spatial characteristics, such as the focal spot morphology and position, show high consistency with both the numerical simulation results (Figure 4c,f) and hydrophone measurements (Figure 4b,e), providing preliminary evidence of LDAQ’s effectiveness in capturing key acoustic field features.

**Figure 5:**
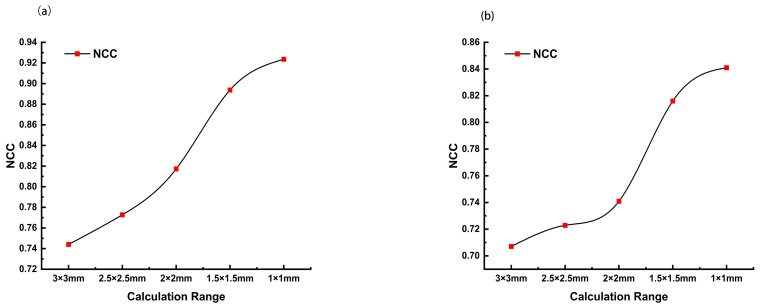
NCC Comparison of LDAQ Model Characteristics with Simulation Results and Inversion Results with Hydrophone Detection Results. (**a**) NCC Comparison between the LDAQ reconstruction results and the simulation results. (**b**) NCC Comparison between the LDAQ reconstruction results and the hydrophone measurement results.

For quantitative assessment, the normalized cross-correlation coefficient (NCC) and root mean square error (RMSE) were calculated between the LDAQ reconstructions and both the simulation and hydrophone results. As shown in Figure 5, the NCC values increased significantly as the evaluation region narrowed toward the central focal zone. In the critical central 1 × 1 mm^2^ focal area, the NCC between LDAQ and the simulation reached 0.924 (Figure 5a),the NCC between LDAQ and the hydrophone measurement results reached 0.843 (Figure 5b), indicating excellent similarity in the core region. In terms of error, the full-field RMSE between LDAQ and simulation was 0.1102, while that between LDAQ and hydrophone measurements was 0.1422, both remaining below 15%, further quantitatively validating the reliability of the method.

**Figure 6:**
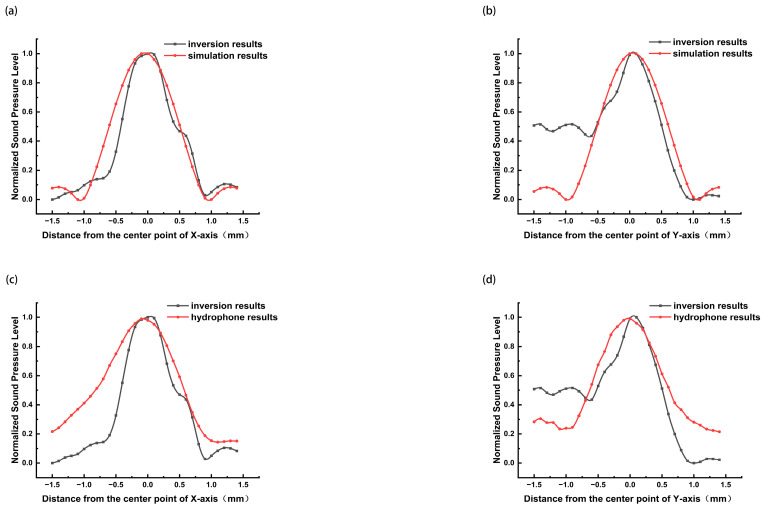
Comparison of the central axis in LDAQ inversion results with simulation results and hydrophone detection results. (**a**,**b**) Comparison of the distribution curves along the X and Y axes between LDAQ sound pressure inversion results and simulation results. (**c**,**d**) Comparison of the distribution curves along the X and Y axes between LDAQ sound pressure inversion results and hydrophone measurement results.

To further examine the detailed agreement, the sound pressure distribution profiles along the central X and Y axes were extracted for comparison. As shown in Figure 6, the curves from the LDAQ inversion and the simulation (Figure 6a,b) nearly coincide in the central region, and also show high overlap with the hydrophone measurement results (Figure 6c,d). This indicates that LDAQ achieved high-fidelity reconstruction of the sound pressure distribution within the focal zone. Some deviations were observed in the peripheral regions, particularly along the Y-axis, potentially due to the amplification of minor experimental perturbations during the inversion process; however, these regions are generally not the primary focus in clinical therapeutic applications.

**Figure 7:**
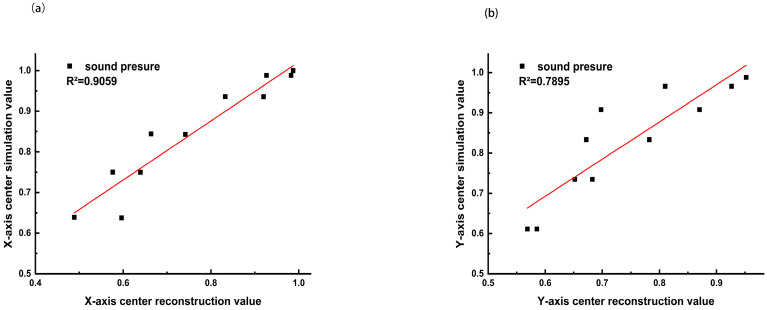
Correlation comparison within the −6dB range of the LDAQ inversion results. (**a**) Correlation coefficient between LDAQ acoustic field inversion results and simulation results in the X-axis direction. (**b**) Correlation coefficient between LDAQ acoustic field inversion results and simulation results in the Y-axis direction.

Finally, the correlation coefficients between the LDAQ-inverted data and the simulation data were calculated within the −6 dB range of the sound pressure peak. As shown in Figure 7, the correlation coefficient along the X-axis direction was as high as 0.9059 (Figure 7a), and that along the Y-axis direction was 0.7895 (Figure 7b), both demonstrating statistically significant correlation and strongly confirming the accuracy of the LDAQ technique for the quantitative characterization of the FU focal zone.

## 4. Discussion

This study successfully developed and validated a non-invasive, high-precision acoustic field quantification method based on the laser deflection principle. Experimental results fully demonstrate that the LDAQ technique can effectively reconstruct the spatial distribution of megapascal-level focused ultrasound fields. The reconstructed results show high consistency with numerical simulations and hydrophone scan data within the focal zone.

The core advantages of the LDAQ technique lie in its non-invasiveness and the high spatial resolution inherent to optical measurement. Compared to conventional hydrophones, it avoids probe-induced distortion of the acoustic field and the risk of damage in high-pressure environments. Compared to complex optical techniques like interferometry, its system structure is relatively simpler, more cost-effective, and easier to deploy. In this study, by employing multi-angle tomographic scanning combined with a Radon transform inversion algorithm, we successfully reconstructed the refractive index gradient distribution of the acoustic field from two coupled physical effects—laser beam intensity variation and spot displacement—and further mapped it to the sound pressure field. The adopted adaptive weighted fusion strategy effectively leveraged the complementary information between intensity and displacement data, enhancing the accuracy and robustness of the reconstruction results.

Quantitative analysis shows that within the central 1 × 1 mm^2^ area of the focal zone, the normalized cross-correlation (NCC) between the LDAQ reconstruction and the numerical simulation reached as high as 0.92355, while the root mean square error (RMSE) across the entire field remained at a low level (0.1102 compared to simulation, 0.1422 compared to hydrophone). This strongly confirms the reliability and accuracy of LDAQ in characterizing the acoustic field within the core region (focal domain) of focused ultrasound. The significant overlap of the sound pressure distribution curves along the central axes further corroborates this point.

Simultaneously, the LDAQ technique, grounded in the fundamental physical effect of acousto-optic deflection, is in principle not limited to the specific transducer configuration or frequency employed in this experiment. The core of this method lies in mapping the optical path deviation caused by the refractive index gradient, which is directly induced by the acoustic pressure gradient. Consequently, it possesses the potential to characterize more complex acoustic fields, such as those at other frequencies, under different focusing conditions, or with non-axisymmetric profiles. The experimental validation presented in this study focuses on a single, canonical focused ultrasound source, aiming to establish the baseline accuracy and feasibility of the LDAQ method. Extending this validation to a broader parameter space remains a crucial objective for future work.

However, this study also observed certain discrepancies between the reconstructed results and the simulations or measurements in the boundary regions of the acoustic field. This may originate from the following factors:

(1) Boundary effects: At the periphery of the acoustic field, the sound pressure gradient changes rapidly. Inversion algorithms (such as the Radon transform) are more sensitive to minor noise and errors in the projection data, potentially leading to partial loss or distortion of boundary information.
(2) Experimental perturbations: Despite vibration isolation measures, minor mechanical vibrations or fluid flow disturbances in the environment might still be detected and amplified by the optical system, particularly in boundary regions with lower sound pressure where the signal-to-noise ratio is relatively reduced.
(3) Model simplification: The numerical simulation involved certain idealizations of the transducer and sound propagation process. Specifically, the concave piezoelectric transducer used in experiments was modeled as an equivalent planar piston source with the same effective aperture. While this simplification is computationally efficient and adequately captures the dominant characteristics of the main focal lobe for the purpose of validating the core LDAQ inversion algorithm, it may not fully replicate the detailed acoustic field structure generated by the curved source. The actual acoustic field might exhibit more complex side-lobes or diffraction effects, and discrepancies arising from aspects not fully captured in the model could lead to differences from experimental results.

Nevertheless, these deviations in the boundary regions have a relatively limited impact on FU therapy. In clinical practice, only the high-energy focal domain is utilized for therapeutic effects such as ablation or neuromodulation, while the peripheral acoustic field, with energy levels orders of magnitude lower, is not targeted and holds minimal clinical relevance. The outstanding performance of the LDAQ technique in focal domain reconstruction provides high-accuracy reconstruction precisely for this therapeutically critical region, enabling reliable extraction of key parameters. Consequently, while the peripheral errors are discussed for methodological completeness, their impact on the assessment of a device’s safety and efficacy profile is fundamentally limited. The outstanding performance of the LDAQ technique in focal domain reconstruction demonstrates its significant application potential for optimizing clinical FU treatment parameters, ensuring treatment safety and efficacy, and promoting the standardization of medical devices.

## 5. Conclusions

To address the quantification needs of focused ultrasound, this study proposes the Laser-Deflected Acoustic Field Quantification (LDAQ) technique. By performing inverse reconstruction on the focused acoustic field generated by a concave piezoelectric ceramic transducer, precise extraction of acoustic field parameters was achieved. Experimental results demonstrate that LDAQ reconstructions exhibit high consistency with numerical simulations and hydrophone measurements in the central acoustic field region: at the focal plane, the normalized cross-correlation coefficient reached 0.92 with an RMS error of 0.11 compared to simulations; while the root mean square error relative to hydrophone measurements was 0.14. This effectively validates the applicability of acousto-optic deflection theory in acoustic field reconstruction and the accuracy of LDAQ technology in practical measurements. This research provides reliable quantitative support for the development of focused ultrasound-based intelligent detection devices and pioneers a non-invasive, cost-effective approach for optical measurement of focused acoustic fields.

## Data Availability

The data presented in this study are available upon reasonable request from the corresponding author. The data are not publicly available due to privacy and ethical reasons.
